# Mining and Characterization of Amylosucrase from *Calidithermus terrae* for Synthesis of α-Arbutin Using Sucrose

**DOI:** 10.3390/ijms252413359

**Published:** 2024-12-12

**Authors:** Anqi Li, Yamei He, Wenxuan Chen, Huimei Tao, Huawei Wu, Shaobin Li

**Affiliations:** College of Life Sciences, Yangtze University, 1 South-Loop Road, Jingzhou 434025, China

**Keywords:** amylosucrase, α-arbutin, enzymatic synthesis, enzymatic properties

## Abstract

α-Arbutin is the fourth generation whitening factor in the field of cosmetics, which can block the synthesis of melanin in epidermal cells and has the advantages of good stability and less toxic side effects. Moreover, α-arbutin has potential application value in food, medicine, and other fields. However, the extraction yield from plant tissues is relatively low, which restricts its application value. Currently, enzymatic catalysis is universally deemed the safest and most efficient method for α-arbutin synthesis. Amylosucrase (ASase), one of the most frequently employed glycosyltransferases, has been extensively reported for α-arbutin synthesis. To discover new resources of amylosucrase (ASase), this study synthesized α-arbutin using low-cost sucrose as a glycosyl donor. Probe sequences were used to identify homologous sequences from different microbial strains in protein databases as candidate ASases. Recombinant plasmids were constructed, and the enzymes were successfully expressed in *Escherichia coli*, followed by the enzymatic synthesis of α-arbutin. One ASase from *Calidithermus terrae*, named CtAs, was selected for its effective α-arbutin synthesis. The expression conditions for CtAs were optimized, its enzymatic properties were analyzed, and the conditions for the enzymatic synthesis of α-arbutin were further refined to improve its molar yield. The optimal induction conditions for CtA expression were achieved by adding IPTG at a final concentration of 0.5 mmol/L to LB medium when OD_600_ reached 1.0, followed by an incubation at 20 °C and 200 r/min for 18 h. The optimal temperature and pH for CtAs were found to be 42 °C and 9.5, respectively, with good stability across the pH range of 5.0–12.0. CtAs was activated by Na^+^, K^+^, Mg^2+^, EDTA, methanol, and ethanol, but inhibited by Ca^2+^, Zn^2+^, Ba^2+^, and Ni^2+^. The kinetic parameters were *V_max_* = 6.94 μmol/min/mL, *K_m_* = 89.39 mmol/L, *K_cat_* = 5183.97 min^−1^, and *K_cat_*/*K_m_* = 57.99 L/(mmol·min). At 42 °C and pH 9.5, the hydrolysis/polymerization/isomerization reaction ratios were 23.27:32.96:43.77 with low sucrose concentrations and 38.50:37.12:24.38 with high sucrose concentrations. The optimal conditions for the enzymatic synthesis were determined to be at 25 °C and pH 5.0 using sucrose at a final concentration of 42 mmol/L and hydroquinone at 6 mmol/L (donor-to-acceptor ratio of 7:1), with the addition of 200 μL (0.2 mg/mL) of purified enzyme and 0.10 mmol/L ascorbic acid, under dark conditions for 6 h. The final molar yield of α-arbutin was 62.78%, with a molar conversion rate of hydroquinone of 74.60%, nearly doubling the yield compared to pre-optimization.

## 1. Introduction

Amylosucrase (ASase) is a glucosyltransferase (EC 2.4.1.4) from the glycoside hydrolase family 13 (GH13) [1]. As a promising multifunctional enzyme, ASase is used in the biosynthesis and modification of starch polysaccharides. In the presence of sucrose, it can catalyze hydrolysis, polymerization, and isomerization reactions, converting sucrose into glucan linked by α-(1,4) glycosidic bonds, while releasing fructose [2,3,4]. In the presence of acceptor molecules, ASase can produce different products, depending on the acceptor, such as amylose-like polysaccharides, oligosaccharides, and transglycosylation products [5,6]. This makes ASase a simple and efficient tool for biosynthesis. However, the development of this enzyme in China is still limited, and its industrial-scale applications remain largely unexplored. It is crucial to discover ASases with excellent catalytic performance, but traditional methods for screening strains are labor-intensive, costly, time-consuming, and often fail to yield ideal results. Therefore, establishing a fast and efficient screening method is equally important.

**Figure 1:**
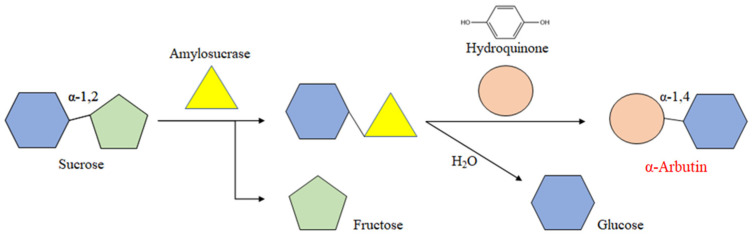
Synthesis of α-arbutin by ASase.

Arbutin [7] (4-hydroquinone-D-glucopyranoside) is a naturally active substance extracted from various plants. It is a glucose-conjugated hydroquinone derivative classified as a hydroquinone glucoside in chemical compounds, and it exhibits specific physiological functions. Arbutin is widely present in animal, plant, and microbial cells [8,9,10]. Studies have shown that α-arbutin has a stronger inhibitory effect on tyrosinase activity, effectively reducing melanin formation without damaging the body [11]. As a whitening agent in cosmetics, α-arbutin has great market potential due to its safety and effectiveness [12,13]. However, the extraction yield from plant tissues is relatively low, which restricts its application value. Previous studies have found that enzymatic synthesis using hydroquinone (HQ) and sucrose as substrates to produce α-arbutin [14,15,16] (Figure 1) offers the advantages of being more efficient, safe, and environmentally friendly. However, there is limited research in China on the production of α-arbutin, and abundant and efficient enzyme resources for this purpose are lacking.

Enzymatic transglycosylation reactions are characterized by their rapid reaction rate, high specificity, and mild reaction conditions [17,18]. Amylosucrase, a typical enzyme involved in transglycosylation, has a wide range of applications [19]. In recent years, with the rise of biosynthetic methods, biosynthesizing α-arbutin using low-cost, pollution-free, and time-efficient approaches has become possible. However, due to the low enzyme production capacity of natural strains and long production cycles, the production costs of the final products are high, hindering the separation and purification of the products [20]. Therefore, the design of recombinant enzymes to increase the enzyme yield and product specificity has become a research focus for both domestic and international researchers. In 2021, researchers identified a novel amylosucrase, Asmet, from a hot spring metagenome, which could use sucrose as a glycosyl donor. At 30 °C and pH 6.0, it achieved a maximum conversion rate of 70% for hydroquinone to α-arbutin after 24 h [15]. In 2023, another study isolated a highly active amylosucrase from haloalkaliphilic bacterium with a yield of 60.3% α-arbutin using 20 mmol/L sucrose and 5 mmol/L hydroquinone as substrates [16].

In this study, we attempted to mine unexplored ASase sequences from different strains with a protein sequence homology ranging from 30% to 85% using biological databases. We selected potential ASase candidates, constructed recombinant plasmids, expressed the proteins, studied their enzymatic properties in order to enrich the resources of transglycosylases, and applied ASases to synthesize α-arbutin, aiming to improve the yield of α-arbutin and provide a new option for the biosynthesis of α-arbutin.

## 2. Results

### 2.1. ASase Mining

**Figure 2:**
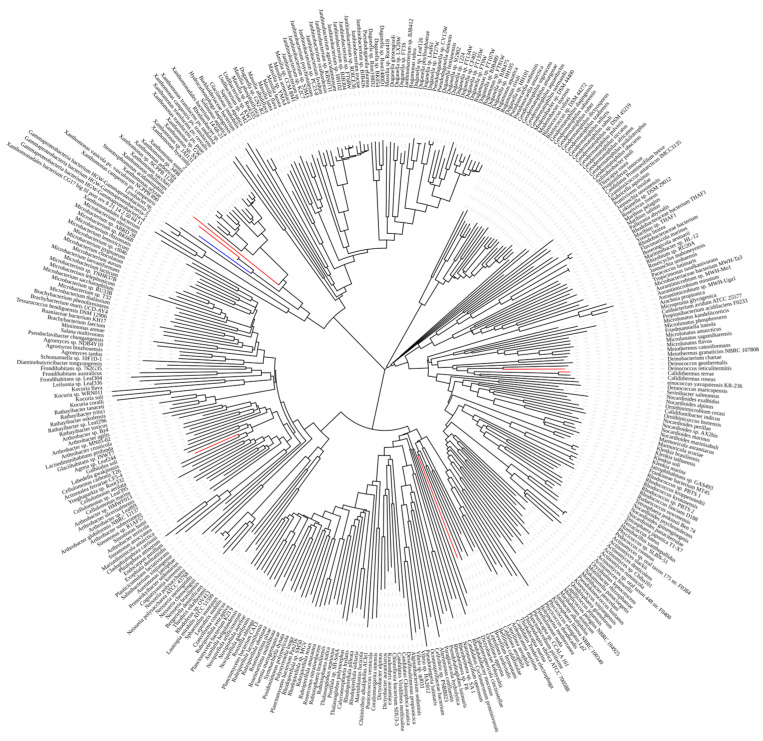
The phylogenetic tree of ASases. (The branches of the probe ASase are marked in blue, and the branches of the candidate ASase enzymes are marked in red.).

ASases with a protein sequence homology of 30–85% were selected, and six potential homologous sequences from different strains were selected as candidate ASases. The phylogenetic tree of all the mined ASase source strains is shown in Figure 2. In the tree, the branches of the probe ASase are marked in blue, and the branches of the candidate ASase enzymes are marked in red. The entire phylogenetic tree is divided into four major evolutionary clades, with the six candidate ASase enzymes distributed among three of these clades. These six ASases were derived from *Xanthomonas oryzae* (GenBank: QBG87180.1) with 78.74% sequence similarity, *Stenotrophomonas acidaminiphila* (GenBank: PZQ29656.1) with 65.61% sequence similarity, *Deinococcus reticulitermitis* (GenBank: SEJ28255.1) with 44.59% sequence similarity, *Calidithermus terrae* (GenBank: RIH84646.1) with 42.42% sequence similarity, *Cellulomonas aerilata* (GenBank: GEO32564.1) with 41.80% sequence similarity, and *Ornatilinea apprima* (GenBank: KPL73089.1) with 36.39% sequence similarity. These ASases were named XoAs, SaAs, DrAs, CtAs, CaAs, and OaAs, respectively.

### 2.2. Phylogenetic Tree Construction and Sequence Comparison and Analysis

**Figure 3:**
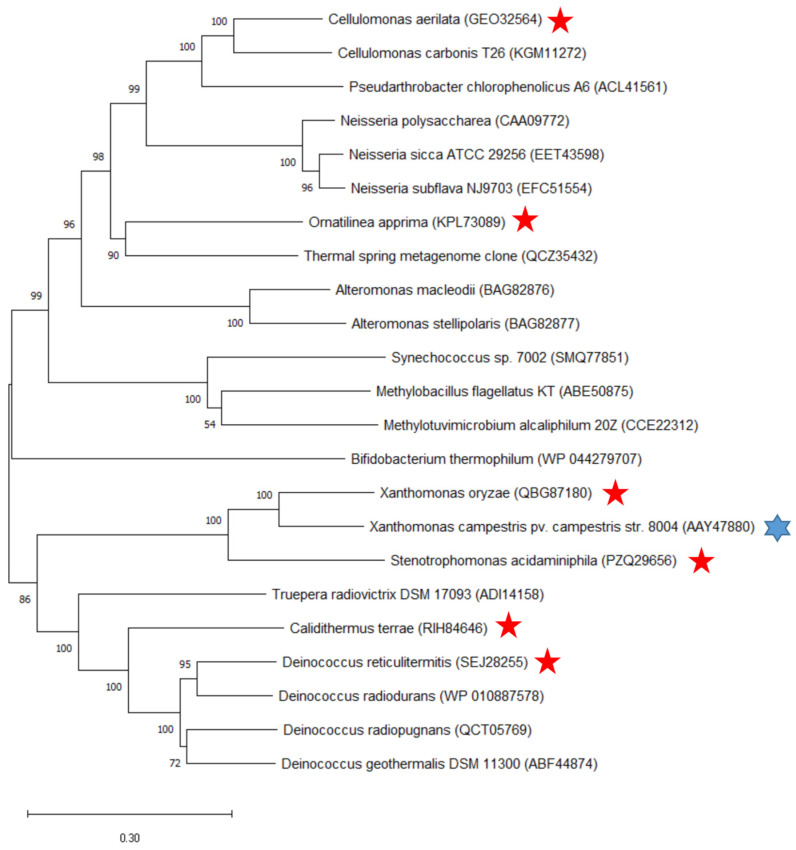
The phylogenetic tree of ASases. (The source strains of the six mined ASase enzymes are marked with red stars, and the source strain of the probe ASase is marked with a blue star).

**Figure 4:**
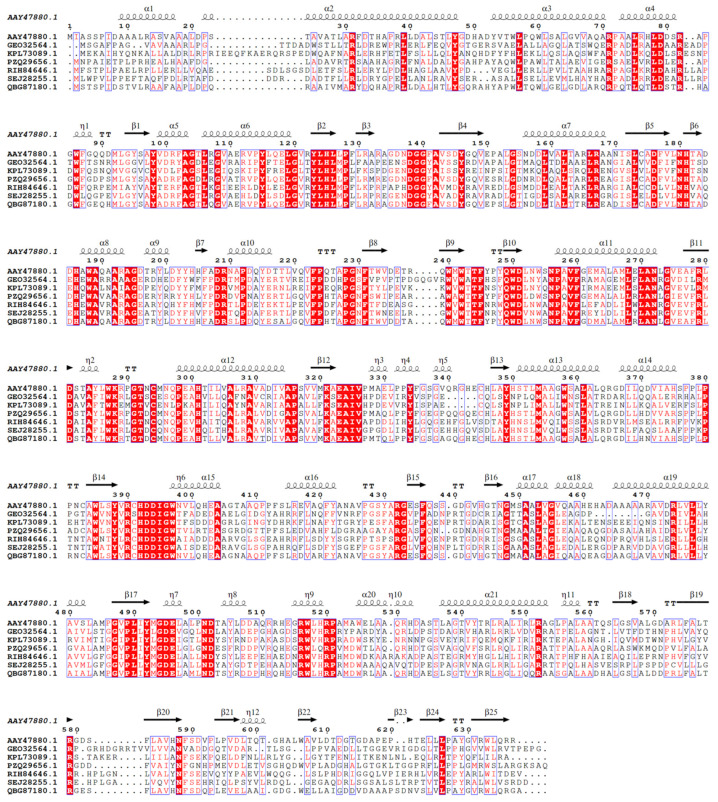
Multiple sequence alignment. (The red background indicates strictly conserved sequences, and the blue boxes indicate highly conserved sequences).

The phylogenetic tree constructed with the six mined ASase enzymes and previously reported ASase enzymes is shown in Figure 3. The phylogenetic tree includes 23 ASase enzymes from 14 genera, and the six candidate ASase enzymes belong to three evolutionary clades. The multiple sequence alignment between the probe sequence and the six mined ASase amino acid sequences is shown in Figure 4. There are 128 strictly conserved amino acids, accounting for about one-fifth of all amino acids, which are key amino acids for ASases, including the core catalytic amino acids such as Asp-281 and Glu-323 [21]. Approximately half of the amino acids are highly conserved, while the others are non-conserved.

### 2.3. Construction of Recombinant ASase Plasmids

Using the synthesized pUC-GW-ASase plasmid as a template, the target genes of the six ASase enzymes were amplified by PCR. The full-length XoAs gene is 1917 bp, encoding 638 amino acids; the full-length SaAs gene is 1929 bp, encoding 642 amino acids; the full-length DrAs gene is 1944 bp, encoding 647 amino acids; the full-length CtAs gene is 1947 bp, encoding 648 amino acids; the full-length CaAs gene is 1920 bp, encoding 639 amino acids; and the full-length OaAs gene is 1947 bp, encoding 648 amino acids.

The target genes of the six ASase enzymes were each ligated into the pET20b(+) vector, resulting in six recombinant plasmids. Sequencing verification confirmed that the sequences were correct.

### 2.4. Induced Expression of ASases

**Figure 5:**
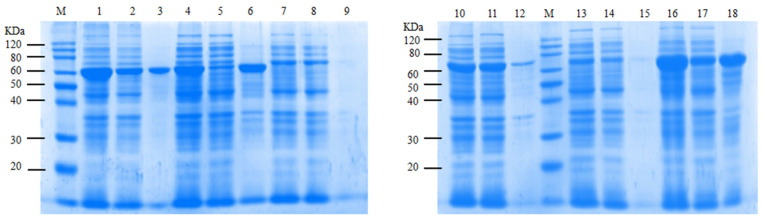
SDS-PAGE gels showing the induced expression of ASases. M: Marker; 1–3: XoAs whole liquid, supernatant, and precipitation suspension; 4–6: SaAs whole liquid, supernatant, and precipitation suspension; 7–9: DrAs whole liquid, supernatant, and precipitation suspension; 10–12: CtAs whole liquid, supernatant, and precipitation suspension; 13–15: CaAs whole liquid, supernatant, and precipitation suspension; and 16–18: OaAs whole liquid, supernatant, and precipitation suspension.

The expression profiles of the six ASase enzymes are shown in Figure 5, with the target protein molecular weight between 70 and 75 kDa. The SDS-PAGE analysis indicated that all six ASases were expressed at varying levels. CtAs showed the best expression, with almost all being soluble. XoAs and OaAs were also highly expressed, although about half was insoluble. SaAs was mostly expressed as an insoluble protein, while DrAs and CaAs had lower expression levels overall.

### 2.5. Screening of ASases for α-Arbutin Enzymatic Synthesis

After the six ASases were induced, purified, and concentrated, the enzyme concentrations were calculated using the bovine serum albumin standard curve. Enzyme activity and specific enzyme activity were calculated based on the fructose standard curve. The enzymatic synthesis reactions were carried out using sucrose and hydroquinone as substrates under certain conditions, and the crude product of α-arbutin was obtained. HPLC analysis was performed, and the molar yield of α-arbutin and the molar conversion rate of hydroquinone were calculated using the α-arbutin standard curve and the hydroquinone standard curve.

The results are shown in Table 1. DrAs, CaAs, and OaAs showed no significant synthesis effects, while CtAs had the highest protein expression (19.22 mg/mL) and exhibited the best catalytic effect on the synthesis reaction, with a molar yield of α-arbutin of 33.79% and a hydroquinone conversion rate of 88.12%. Thus, CtAs was selected for further experiments.

### 2.6. Optimization of Soluble Expression Conditions for CtAs

**Figure 6:**
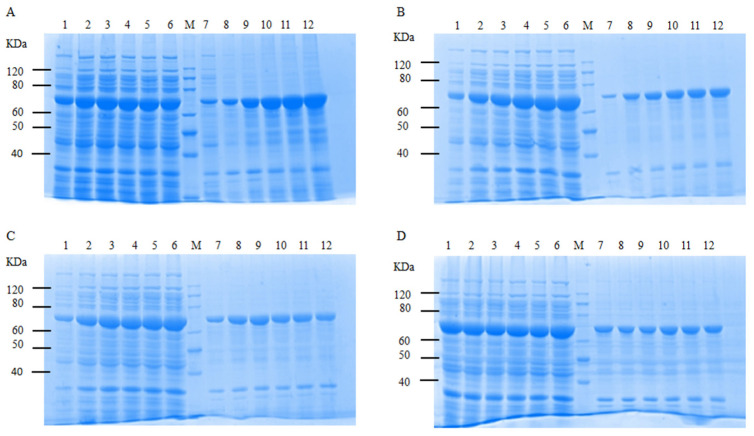
Optimization of soluble expression conditions for CtAs by SDS-PAGE electrophoresis. (**A**) Induction temperature, 1–6: 15, 20, 25, 30, 35, and 40 °C; supernatant, 7–12: 15, 20, 25, 30, 35, and 40 °C; precipitation suspension; (**B**) induction time, 1–6: 2, 6, 10, 14, 16, 18, and 22 h; supernatant, 7–12: 2, 6, 10, 14, 16, 18, and 22 h; precipitation suspension; (**C**) induction time: 1–6: OD_600_ = 0.2, 0.4, 0.6, 0.8, 1.0, and 1.2; supernatant, 7–12: OD_600_ = 0.2, 0.4, 0.6, 0.8, 1.0, and 1.2; precipitation suspension; and (**D**) IPTG addition amount: 1–6: 0.1, 0.3, 0.5, 0.7, 0.9, and 1.1 mmol/L; supernatant, 7–12: 0.1, 0.3, 0.5, 0.7, 0.9, and 1.1 mmol/L; precipitate suspension; M: marker.

To determine the optimal conditions for soluble expression of CtAs, single-factor optimizations were performed on the induction temperature, time, point of induction, and IPTG concentration. The SDS-PAGE analysis of the crude enzyme solutions under various conditions is shown in Figure 6. As the induction temperature increased, the amount of soluble protein decreased, with the highest soluble protein expression and the lowest insoluble protein expression at 20 °C (Figure 6A). With increasing induction time, both the soluble and insoluble protein amounts increased, with the best soluble expression and minimal insoluble expression occurring at 18 h (Figure 6B). As the OD_600_ increased, the amount of soluble protein also increased, with the best expression achieved at an OD_600_ of 1.0, where the amount of insoluble protein decreased (Figure 6C). The amount of soluble protein expression did not change significantly with increasing IPTG concentrations, but insoluble protein expression increased noticeably when IPTG concentrations exceeded 0.5 mmol/L (Figure 6D).

Therefore, the optimal conditions for soluble expression of CtAs were determined to be the addition of 0.5 mmol/L IPTG at an OD_600_ of 1.0, with induction at 20 °C for 18 h.

**Figure 7:**
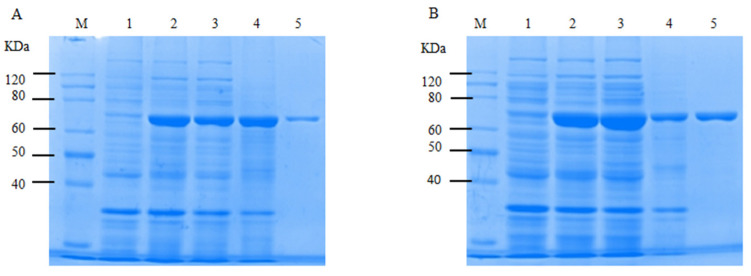
Comparison of SDS-PAGE gels before and after the optimization of CtAs soluble expression conditions. (**A**) Soluble expression status before the optimization of CtAs. (**B**) Soluble expression status after the optimization of CtAs. M: Marker; 1: empty control; 2: whole liquid; 3: supernatant; 4: precipitation suspension; and 5: purified enzyme solution.

After the purification and ultrafiltration concentration of the crude CtAs enzyme solution, the purified enzyme was obtained. A comparison of pre- and post-optimization expression is shown in Figure 7. Post-optimization expression (Figure 7B) was significantly increased in the supernatant and whole solution, while insoluble expression in the precipitate decreased significantly. The supernatant, after purification, yielded a pure single target protein.

### 2.7. Analysis of the Enzymatic Properties of CtAs

**Figure 8:**
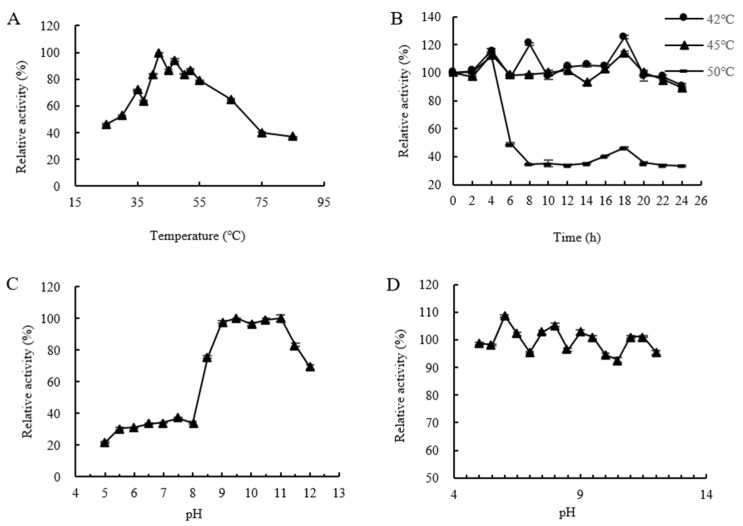
Analysis of the enzymatic properties of CtAs. (**A**) The optimal temperature of CtAs. (**B**) The thermal stability of CtAs. (**C**) The optimal pH of CtAs. (**D**) The pH stability of CtAs. (Values are the means of three replicates ± standard deviations).

The enzymatic properties of CtAs were analyzed, as shown in Figure 8. The optimal temperature for CtAs was 42 °C, with the relative enzyme activity remaining above 80% between 40 °C and 55 °C, but decreasing when the temperature exceeded 55 °C (Figure 8A). Under conditions of 42 °C and 45 °C, the relative enzyme activity remained above 80% for 24 h and above 90% for 18 h, indicating good thermal stability. At 50 °C, the relative enzyme activity dropped below 50% after 4 h (Figure 8B).

The optimal pH for CtAs was 9.5, and it was stable within a pH range of 9.0–11.0, maintaining a relative enzyme activity above 95%. At pH values below 8.5, the relative enzyme activity dropped below 40% (Figure 8C). CtAs remained stable across a pH range of 5.0–12.0, with relative enzyme activity above 90%, indicating broad pH stability (Figure 8D).

The effects of various metal ions and reagents on CtAs enzyme activity are shown in Table 2. Mn^2+^, Cu^2+^, Fe^2+^, and SDS activated CtAs at low concentrations (0.2 and 1 mmol/L). Except for 5 mmol/L Fe^2+^, which had little effect, higher concentrations of other ions (5 and 10 mmol/L) inhibited CtAs activity. Na^+^, K^+^, Mg^2+^, and EDTA activated CtAs at both low and high concentrations, while Ca^2+^, Zn^2+^, Ba^2+^, and Ni^2+^ inhibited CtAs at both low and high concentrations.

The effects of organic solvents on CtAs activity are shown in Table 3. n-Butanol activated CtAs at low concentrations (1% and 5%) but inhibited its activity at high concentrations (10% and 20%). Methanol activated CtAs at both low and high concentrations. Ethanol inhibited CtAs at 20%, while acetonitrile activated CtAs at 1%. Dimethyl sulfoxide had little effect at low concentrations, but at 20%, it inhibited CtAs activity to some extent.

**Figure 9:**
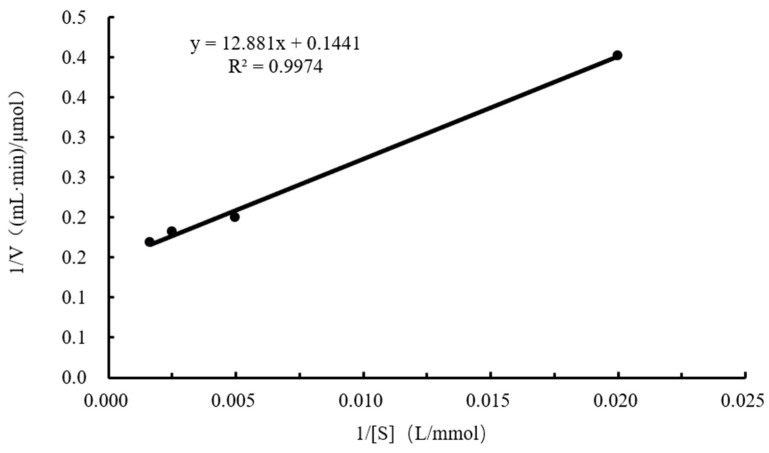
Kinetic curve.

The kinetic curve is shown in Figure 9. Based on the Lineweaver–Burk plot, the kinetic parameters were calculated as *V_max_* = 6.94 μmol/min/mL, *K_m_* = 89.39 mmol/L, *K_cat_* = 5183.97/min, and *K_cat_*/*K_m_* = 57.99 L/(mmol·min).

**Figure 10:**
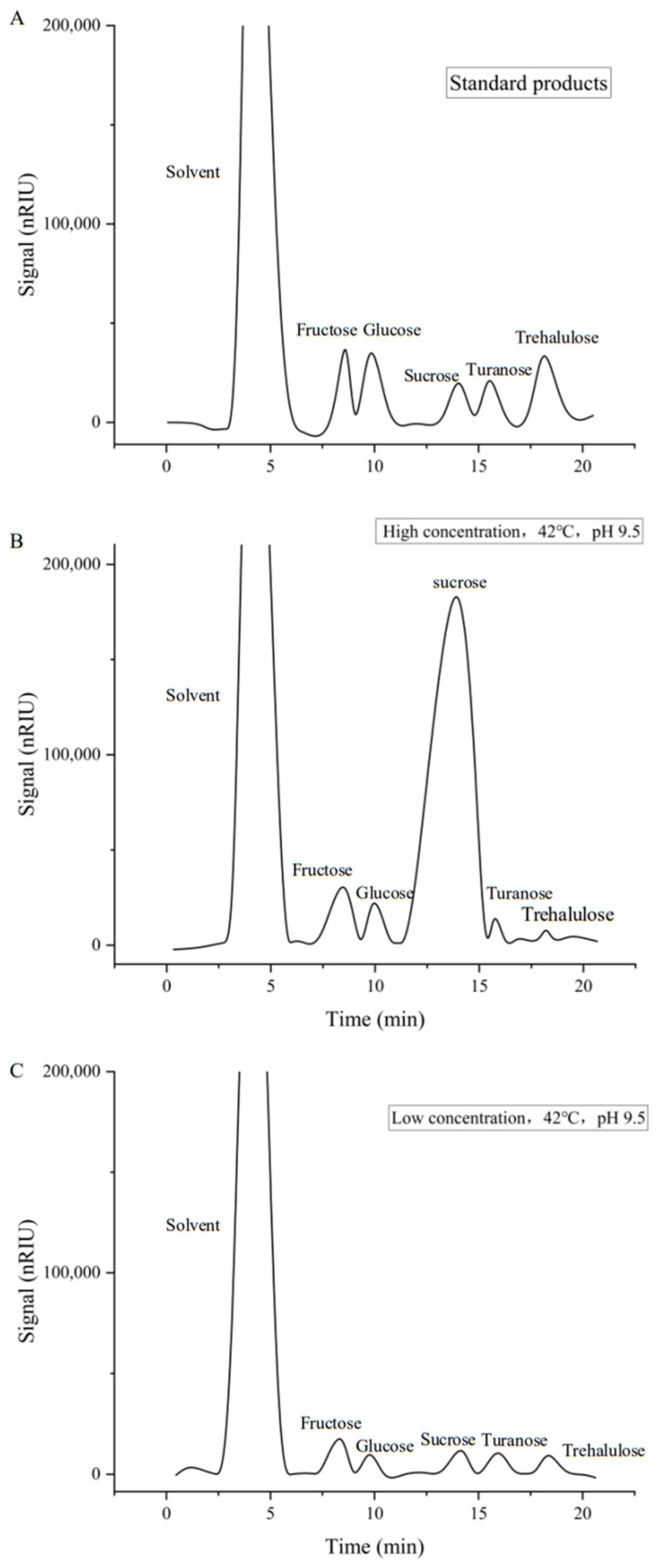
Analysis of reaction products by HPLC. ((**A**) The chromatographic peaks from the HPLC analysis of standard products; (**B**) The chromatographic peaks from the HPLC analysis at low sucrose concentration, 45 °C, pH9.5 reaction conditions; (**C**) The chromatographic peaks from the HPLC analysis at high sucrose concentration, 45 °C, pH9.5 reaction conditions).

The chromatographic peaks from the HPLC analysis of reactions with sucrose as the sole substrate are shown in Figure 10. Based on the peak area calculations, the reaction ratios at 42 °C and pH 9.5 were hydrolysis/polymerization/isomerization = 23.27:32.96:43.77 for low sucrose concentrations, and hydrolysis/polymerization/isomerization = 38.50:37.12:24.38 for high sucrose concentrations. Low sucrose concentrations favored isomerization reactions, while high sucrose concentrations favored hydrolysis reactions, with polymerization reactions remaining relatively unaffected. Both isomerization and polymerization are transglycosylation reactions, and when other acceptors are present, transglycosylation occurs, forming other glycosyl derivatives. Under the optimal conditions for CtAs, the reaction ratios for hydrolysis/transglycosylation were 23.27:76.73 with low sucrose concentrations and 38.50:61.50 with high sucrose concentrations. In both cases, transglycosylation predominated, with the ratio being higher with low sucrose concentrations.

### 2.8. Optimization of α-Arbutin Enzymatic Synthesis Conditions

**Figure 11:**
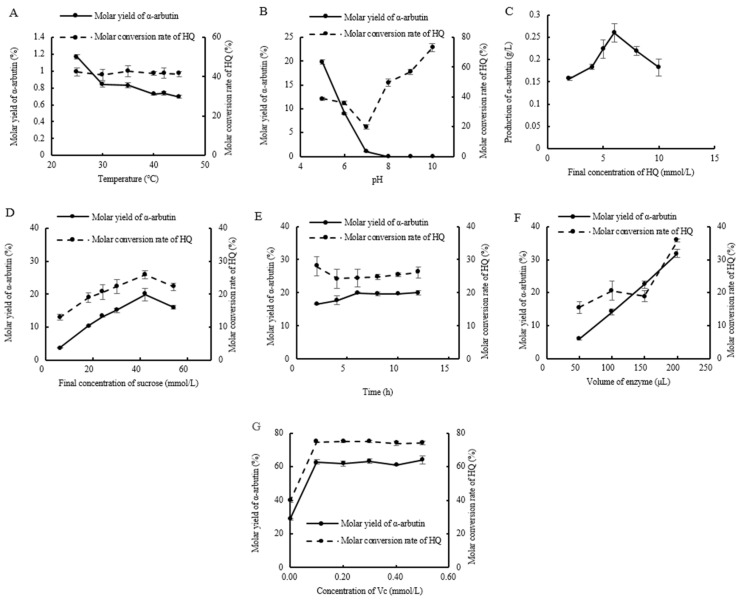
Optimization of α-arbutin synthesis conditions. ((**A**) Temperature; (**B**) pH; (**C**) Final concentration of HQ; (**D**) Final concentration of sucrose; (**E**) Time; (**F**) Volume of enzyme; (**G**) Concentration of Vc.) (Values are the means of three replicates ± standard deviations).

As shown in Figure 11, the molar yield of α-arbutin decreased as the temperature increased, while the molar conversion rate of hydroquinone remained nearly constant at around 40%. From this, we can conclude that the optimal reaction temperature was 25 °C (Figure 11A). As the pH increased, the molar yield of α-arbutin dropped rapidly, and synthesis almost ceased at pH levels above 7. The molar conversion rate of hydroquinone first decreased and then increased, and so the optimal pH was 5.0 (Figure 11B). When varying the final concentration of hydroquinone, the optimal concentration was 6 mmol/L. However, when the concentration exceeded 6 mmol/L, the yield of α-arbutin gradually decreased (Figure 11C). The optimal sucrose concentration was 42 mmol/L, with a donor-to-acceptor ratio of 7:1. And when this ratio exceeded 7:1, further increases in the sucrose concentration led to a decrease in the molar yield of α-arbutin (Figure 11D). The optimal reaction time was 6 h, after which the molar yields of both α-arbutin and hydroquinone stabilized (Figure 11E). As the amount of enzyme increased, both the molar yield of α-arbutin and the molar conversion rate of hydroquinone increased, with the optimal enzyme dose being 200 μL (0.2 mg/mL) (Figure 11F). Additionally, when a certain amount of vitamin C (Vc) was added to inhibit the oxidation of hydroquinone, it was found that 0.10 mmol/L Vc greatly enhanced both the molar yield of α-arbutin and the molar conversion rate of hydroquinone. Further increases in the Vc concentration had little effect on the reaction efficiency (Figure 11G). Ultimately, the molar yield of α-arbutin reached 62.78%, and the molar conversion rate of hydroquinone was 74.60%, nearly doubling compared to the pre-optimization results.

## 3. Discussion

The six different ASases obtained through mining were identified more conveniently and quickly using a large computer database compared to traditional strain screening methods. The phylogenetic tree showed that the six ASases were distributed across different branches, and their evolutionary relationships varied compared to previously reported ASases, reflecting diversity in strain sources.

In the optimization of soluble expression conditions for CtAs, it was found that low-temperature induction increased soluble expression, consistent with previous studies. Temperature significantly affects *E. coli* growth, plasmid stability, the recombinant protein expression rate, and protein folding efficiency [22,23]. Protein aggregation is primarily driven by interactions between exposed hydrophobic structures, and the temperature dependence of hydrophobic interactions dictates the rate of aggregation. Higher temperatures accelerate protein aggregation [24], leading to the formation of insoluble inclusion bodies. Lower temperatures reduce *E. coli* proliferation, transcription, and translation rates, resulting in lower protein expression [25]. However, slower translation rates promote proper protein folding, reducing aggregation and enhancing soluble recombinant protein expression [26]. IPTG competitively binds to the lac repressor, initiating the transcription of the target protein. Excessive IPTG concentrations lead to the over-activation of transcription and protein translation, causing the excessive accumulation of translation precursors and promoting inclusion body formation. Conversely, insufficient IPTG concentrations fail to effectively bind to the lac repressor [27], affecting protein expression levels.

In the analysis of the enzymatic properties of CtAs, a comparison of the enzymatic properties of recombinant ASases from different strains is shown in Table 4. The optimal temperature of CtAs in this study falls within the typical range for ASases (30–50 °C), and it has a higher optimal temperature than five of the ASases. Its optimal pH of 9.5 is higher than that of all currently reported ASases, and it remains stable within a pH range of 5.0–12.0. Among the reported ASase reaction proportions, polymerization reactions typically dominate, followed by isomerization reactions, with hydrolysis reactions being the least common. However, in this study, isomerization reactions were the most dominant, while polymerization reactions were lower, and hydrolysis reactions were higher than those of other ASases. As in other ASase studies, the reaction proportions vary with temperature, pH, and substrate concentration, but there is no consistent pattern [1,16,28].

The enzymatic synthesis of α-arbutin after optimization showed significant advantages compared to other studies using ASases for α-arbutin synthesis. Seo et al. [41] used a constitutive expression system to express and purify ASase from *Deinococcus geothermalis* (DGAS) in *E. coli*, achieving an α-arbutin conversion rate of only 1.3%. In contrast, this study achieved a molar conversion rate of 31.85% for α-arbutin without adding Vc, with CtAs performing better than DGAS. After adding a small amount of Vc, the instability of hydroquinone was greatly improved. In the presence of 0.2 mmol/L Vc, when the donor (sucrose), acceptor (hydroquinone), and Vc were reacted at a molar ratio of 10:1:0.1 (23:2.3:0.2 mmol/L), the hydroquinone conversion rate was 90%. In comparison, this study achieved a hydroquinone conversion rate of 74.60% after adding Vc, which is lower than the DGAS conversion rate, possibly due to differences in substrate concentrations. Yu et al. [42] used *Cellulomonas carboniz* T26 CcAS for the synthesis of α-arbutin, using 20 mmol/L sucrose and 5 mmol/L hydroquinone as the donor and acceptor. Under the optimal conditions of pH 7.0 and 40 °C for 2 h without adding Vc, the molar yield of α-arbutin reached 40–44.7%. With high hydroquinone concentrations, CcAS achieved a molar yield of 16.1% without the addition of ascorbic acid. Compared to this study, the yield without Vc was higher, while the yield with Vc was lower, and higher yields were achieved with low hydroquinone concentrations.

CtAs is a promising ASase with a high molar yield of α-arbutin, indicating its potential application in industrial production. It provides a new option for the industrial-scale production of α-arbutin.

## 4. Materials and Methods

### 4.1. Materials

The pET20b(+) plasmid used to construct recombinant plasmids was preserved in our laboratory. *E. coli* DH5α was used as the host strain for plasmid construction, while *E. coli* BL21(DE3) was used as the host strain for gene expression. The inorganic reagents and organic solvents used were all of analytical grade from local manufacturers. The restriction enzymes, EasyTAQ^®^ DNA polymerase, and T4 DNA ligase used in the experiments were purchased from TAKARA (Dalian, China).

LB liquid medium: A total of 5.0 g of yeast extract, 10.0 g of tryptone, and 10.0 g of sodium chloride were dissolved in 1 L of deionized water. The pH was adjusted to 7.0–7.2 with NaOH and sterilized at 121 °C for 20 min.

LB solid medium: Chemically pure agar powder was added to the LB liquid medium at a ratio of 1.0–2.0 g agar per 100 mL of LB medium, and then sterilized at 121 °C for 20 min.

### 4.2. Methods

#### 4.2.1. Exploration of ASases

A previously reported ASase with a high α-arbutin synthesis rate, Amy-1, from *Xanthomonas campestris* pv. *campestris* (strain 8004) (GenBank: AAY47880.1) [43,44] was used as a probe. Its amino acid sequence was retrieved from the NCBI database (https://blast.ncbi.nlm.nih.gov/Blast.cgi, accessed on 12 December 2024). The amino acid sequence of Amy-1 was input into the Protein Blast online tool on the UniProt database (https://www.uniprot.org/blast, accessed on 12 December 2024) to search for homologous sequences. To ensure the diversity and coverage of the mined ASase enzymes, bacterial ASase sequences with homology to the probe ranging from 30% to 85% were selected to construct a phylogenetic tree. Based on the source and evolutionary relationships, six homologous sequences from different strains were selected as candidate ASase enzymes.

#### 4.2.2. Construction of the Phylogenetic Tree and Sequence Comparison and Analysis

Using MEGA 11 software (https://megasoftware.net/, accessed on 12 December 2024) and the neighbor-joining method, a phylogenetic tree was constructed with the six ASase enzymes and previously reported ASase source strains. The analysis was set to perform 1000 bootstrap replicates to assess the evolutionary relationships between the strains. The online program ESPRIPT3 (https://espript.ibcp.fr/ESPript/cgi-bin/ESPript.cgi, accessed on 12 December 2024) was used for the multiple sequence alignment analysis between the probe sequence and the six ASase enzymes to assess sequence conservation.

#### 4.2.3. Construction of Recombinant ASase Plasmids

The nucleotide sequences of the six candidate ASase enzymes were synthesized by GenScript Biotech (Suzhou, China), and primers were designed, as shown in Table 5. The pUC-GW-ASase plasmid synthesized by the company was used as a template for the PCR amplification of the target genes. The PCR program was as follows: 95–98 °C for 5 min; 95–98 °C for 30 s, 55–65 °C for 30 s (adjusted according to the T_m_ of different primers), 72 °C for 2 min, repeated for 30 cycles; 72 °C for 10 min; and then a hold at 4 °C. The target gene was recovered from the gel using the Omega Gel Extraction Kit, and plasmid extraction was performed using the Plasmid Miniprep Kit following the manufacturer’s instructions. Both the gel-purified target gene and the pET20b(+) plasmid were digested with restriction enzymes to produce compatible sticky ends. The digested target gene and pET20b(+) plasmid were ligated to construct the recombinant plasmid. The correctly verified recombinant plasmids were sent for sequencing, and the correctly sequenced six recombinant plasmids were transformed into *E. coli* BL21(DE3) competent cells by heat shock, followed by culturing the recombinant expression strains and storing them at −80 °C.

#### 4.2.4. Induced Expression of ASases

The overnight cultures of the recombinant expression strains were used as seed cultures. The seed cultures were inoculated into LB liquid medium containing 100 μg/mL ampicillin at a 1% inoculum ratio, and cultured at 37 °C and 200 r/min until the OD_600_ reached 0.6. IPTG was added at a final concentration of 0.5 mmol/L, and induction was performed at 30 °C and 200 r/min for 6 h. The bacterial cultures were collected by centrifugation at 12,000 r/min at 4 °C for 10 min. The bacterial pellets were washed twice with 50 mmol/L PBS buffer (pH 7.4) and then resuspended in the buffer. The bacterial cells were disrupted using an ultrasonic homogenizer at 75% power (total power 500 W), with 3 s on and 6 s off, in an ice bath for 15–30 min until the solution became clear. This resulted in the crude enzyme solution. The crude enzyme solution was centrifuged at 10,000 r/min at 4 °C for 20 min to collect the supernatant, which was used as the crude enzyme extract. The pellet was also resuspended in 50 mmol/L PBS (pH 7.4) buffer, yielding the pellet suspension. Protein expression was analyzed using SDS-PAGE with a 10% separating gel and 5% stacking gel. Samples were mixed with loading buffer, boiled for 10 min, centrifuged at 8000 r/min for 8 min, and 10 μL of the supernatant was loaded along with 5 μL of the marker. Electrophoresis was performed at 80 V for 30 min, followed by 120 V for approximately 1.5 h. The gel was stained with Coomassie Brilliant Blue R250 and decolorized. The results were observed using a gel imaging system. Crude enzyme extracts were purified using Ni-NTA His Bind Resin metal-affinity chromatography. The specific operations followed the manufacturer’s instructions for the purification column. Target proteins were eluted with 200 mmol/L imidazole buffer, and the eluted proteins were desalted and concentrated using Millipore ultrafiltration centrifuge tubes at 5000 r/min at 4 °C for 40 min, yielding purified enzymes, which were stored at 4 °C.

#### 4.2.5. Screening of ASases for the Enzymatic Synthesis of α-Arbutin

The concentrations of the purified enzymes were determined using the Bradford method [44]. For the bovine serum albumin standard curve, a 1 mg/mL bovine serum albumin stock solution was prepared in 0.01 mol/L PBS buffer (pH 7.4). Serial dilutions were made to obtain different concentrations of bovine serum albumin (0.20, 0.16, 0.12, 0.08, 0.04 mg/mL). A total of 30 μL of each concentration was mixed with 1 mL of G-250 solution, incubated for 10 min, and the absorbance was measured at 595 nm, using a blank without protein for zeroing. The standard curve was plotted with the protein concentration on the *x*-axis and absorbance on the *y*-axis.

Following the method of Seo et al. [41], enzyme activity was determined using the dinitrosalicylic acid (DNS) method. One unit of enzyme activity was defined as the amount of enzyme required to catalyze the production of 1 μmol of fructose per minute under conditions of 30 °C and pH 7.0. For the fructose standard curve, a 1 mg/mL fructose stock solution was prepared and diluted to various concentrations (100, 120, 140, 160, 180, and 200 μg/mL). A total of 400 μL of each concentration was mixed with 600 μL of DNS solution, heated in a boiling water bath for 15 min, and then cooled. The absorbance was measured at 540 nm. The standard curve was plotted with the fructose concentration on the *x*-axis and absorbance on the *y*-axis.

The reaction system for samples consisted of 250 μL of 100 mmol/L Tris-HCl (pH 7.0), 100 μL of a 4% sucrose solution, and 50 μL of the enzyme solution (diluted as needed). The reaction was carried out at 30 °C for 10 min, followed by the addition of 600 μL of the DNS solution to terminate the reaction. After a 15 min incubation in a boiling water bath, the samples were cooled, and the absorbance was measured at 540 nm. Each reaction was performed in triplicate using heat-inactivated enzyme as a control and a blank without enzyme for zeroing.

Six ASase enzymes were used for the enzymatic synthesis reaction, and the one with the best α-arbutin synthesis performance was selected. Following the method of Agarwal et al. [15], the reaction system was set up as follows: 0.30 mL of 5 mmol/L hydroquinone, 0.30 mL of 20 mmol/L sucrose, equal amounts of purified enzyme, and 50 mmol/L phosphate buffer (pH 7.0) to make up 1 mL. The mixture was incubated in the dark at 30 °C for 2 h, and the reaction was terminated by boiling for 10 min. The supernatant was obtained by centrifugation at 12,000 r/min at 4 °C for 20 min and filtered through a 0.22 μm aqueous microporous membrane. The enzymatic products were analyzed using high-performance liquid chromatography (HPLC).

HPLC conditions: Waters E2698 HPLC, Inertsil ODS-3 C18 column (5 μm, 4.60 mm × 250 mm), PDA detector, mobile phase of 5% methanol and 95% water, flow rate of 1 mL/min, column temperature of 25 °C, injection volume of 10 μL, and detection wavelength of 282 nm.

For the α-arbutin standard curve, a 1.6 mg/mL α-arbutin stock solution was prepared and serially diluted to different concentrations (1.6, 1.4, 0.8, 0.4, 0.2, 0.1, 0.05, and 0.025 mg/mL). For the hydroquinone standard curve, a 16 mg/mL hydroquinone stock solution was prepared and serially diluted to different concentrations (1.6, 1.4, 0.8, 0.4, 0.2, 0.1, 0.05, and 0.025 mg/mL). The peak area was plotted on the y-axis, and the concentration on the x-axis to create the standard curves. Based on the peak areas measured from the samples, the molar yield of α-arbutin and the molar conversion rate of hydroquinone were calculated using the standard curves. The calculation formulas are as follows:

For the α-arbutin molar yield (%),

(1) $$C=\frac{n_{0}}{n_{1}}\times 100\%$$

where C is the molar conversion rate of α-arbutin, *n*_0_ is the molar yield of α-arbutin (mmol), and *n*_1_ is the molar amount of hydroquinone before the reaction (mmol).

For the hydroquinone molar conversion rate (%),

(2) $$C=\frac{n_{1}-n_{2}}{n_{1}}\times 100\%$$

where C is the molar conversion rate of hydroquinone, *n*_1_ is the molar amount of hydroquinone before the reaction (mmol), and *n*_2_ is the molar amount of hydroquinone remaining after the reaction (mmol).

#### 4.2.6. Optimization of the Soluble Expression Conditions for CtAs

To improve the soluble expression yield of CtAs, single-factor optimization was performed on the induction temperature, induction time, induction point, and IPTG concentration. The recombinant expression strain of CtAs was inoculated into 5 mL of LB liquid medium containing 100 μg/mL Ampicillin was added at a 2% inoculum ratio and bacteria were cultured overnight at 37 °C and 200 r/min as a seed culture. The seed culture was transferred to 25 mL of LB liquid medium containing 100 μg/mL ampicillin at a 1% inoculum ratio and cultured at 37 °C and 200 r/min until the OD_600_ reached 0.6, at which point IPTG was added at a final concentration of 0.5 mmol/L. Expression was induced at different temperatures (15, 20, 25, 30, 35, or 40 °C) for 6 h to determine the optimal induction temperature.

Keeping the other conditions constant, the induction time was optimized by inducing expression at the optimal temperature for different durations (0, 6, 10, 14, 18, or 22 h).

Keeping the other conditions constant, IPTG was added at different OD_600_ values (0.2, 0.4, 0.6, 0.8, 1.0, or 1.2) to determine the optimal induction point.

Finally, IPTG was added at different concentrations (0.1, 0.3, 0.5, 0.7, 0.9, or 1.1 mmol/L) at the optimal temperature, time, and cell density to determine the optimal IPTG concentration. SDS-PAGE was used to analyze protein expression, with the empty plasmid as a control, and the best expression conditions were selected.

#### 4.2.7. Analysis of Enzymatic Properties 

The method for determining enzyme activity is the same as described in Section 4.2.5.

(1) Optimal temperature for CtAs: The reaction system was incubated at different temperatures (25–85 °C) for 10 min, and enzyme activity was measured. The highest enzyme activity was set as 100%, and the relative enzyme activity was calculated to determine the optimal reaction temperature for CtAs.
(2) Thermal stability of CtAs: The enzyme solution was mixed with 100 mmol/L Tris-HCl (pH 7.0) and incubated at 40–50 °C for 0–24 h. Afterward, a 4% sucrose solution was added, and the reaction was carried out at the optimal temperature for 10 min. The remaining enzyme activity was measured, with the untreated enzyme activity set to 100%. The relative enzyme activity was calculated to determine the thermal stability of CtAs.
(3) Optimal pH of CtAs: The reaction system was incubated with 100 mmol/L Tris-HCl buffer at different pH values (5.0–12.0) at the optimal temperature for 10 min, and enzyme activity was measured. The highest enzyme activity was set to 100%, and the relative enzyme activity was calculated to determine the optimal pH for CtAs.
(4) pH stability of CtAs: The enzyme solution was mixed with 100 mmol/L Tris-HCl buffer at different pH values (5.0–12.0) and incubated at 25 °C for 1 h. The reaction was then carried out at the optimal temperature and pH for 10 min, and the remaining enzyme activity was measured. The untreated enzyme activity was set to 100%, and the relative enzyme activity was calculated to determine the pH stability of CtAs.
(5) Metal ion and organic solvent stability of CtAs: The reaction system was supplemented with metal ions and reagents (Na^+^, K^+^, Mg^2+^, Ca^2+^, Zn^2+^, Ba^2+^, Mn^2+^, Ni^2+^, Cu^2+^, Fe^2+^, EDTA, and SDS) at final concentrations of 0.2, 1, 5, or 10 mmol/L, or organic solvents (methanol, ethanol, n-butanol, acetonitrile, and dimethyl sulfoxide) at final concentrations of 1%, 5%, 10%, or 20%. The reaction was carried out at the optimal temperature and pH for 10 min, and enzyme activity was measured. The enzyme activity in a system without metal ions or organic solvents was set to 100%, and the relative enzyme activity was calculated to determine the effects of metal ions and organic solvents on CtAs activity.
(6) Kinetic parameters: Different concentrations of the sucrose solution (0.05, 0.2, 0.4, and 0.6 mol/L) were prepared in 100 mmol/L Tris-HCl buffer (pH 7.0) and added to the reaction system. Enzyme activity was measured at the optimal temperature and pH for each substrate concentration. The Lineweaver–Burk plot was used to calculate the kinetic parameters *K_cat_*, *K_m_*, and *K_cat_*/*K_m_*. The reciprocal of the sucrose concentration was used as the abscissa, and the reciprocal of enzyme activity was plotted as the ordinate.
  The calculation formula is as follows:
  (3) $$\frac{1}{V}=\frac{K_{m}}{V_{max}}\times \frac{1}{\left[S\right]}+\frac{1}{V_{max}}$$
  (4) $$K_{cat}=\frac{V_{max}}{\left[E\right]}$$
(7) Enzyme reaction proportion: The reaction system (low sucrose concentration of 89.39 mmol/L and high sucrose concentration of 893.90 mmol/L) was incubated at the optimal temperature and pH for 2 h. The reaction was terminated by boiling for 5 min, centrifuged at 12,000 r/min for 20 min at 4 °C, and filtered through a 0.22 μm microporous membrane. The products were analyzed using HPLC. Using the method of Huang Liangjiang et al. [45,46,47], the HPLC conditions were as follows: Agilent 1200 HPLC (Agilent Technologies, Waldbronn, Germany), ZORBAX NH2 column (5 μm, 4.60 mm × 250 mm), RID detector, mobile phase of 80% acetonitrile and 20% water, flow rate of 1 mL/min, column temperature of 35 °C, detector chamber of 40 °C, room temperature of 30 °C, and manual injection volume of 20 μL. Standard solutions of fructose, glucose, sucrose, maltose, and trehalose (10 mg/mL) were prepared. Based on the peak area results, the percentage of each reaction type was calculated relative to the total peak area, and the catalytic properties of ASases were analyzed.

#### 4.2.8. Optimization of α-Arbutin Enzymatic Synthesis Conditions

To improve the molar yield and production of α-arbutin via enzymatic synthesis, the effects of the reaction temperature, pH, reaction time, final concentrations of hydroquinone and sucrose, enzyme dose, and the addition of ascorbic acid (Vc) were investigated to determine the optimal reaction conditions. The reaction system consisted of 0.40 mL of hydroquinone, 0.40 mL of sucrose, an appropriate amount of the purified enzyme solution (1 mg/mL), and 50 mmol/L phosphate buffer to make up 1 mL. The reaction was carried out in the dark at specific temperatures for set durations and terminated by boiling for 10 min. After centrifugation at 12,000 r/min for 20 min at 4 °C, the supernatant was filtered through a 0.22 μm microporous membrane. The α-arbutin molar yield and hydroquinone molar conversion rate were determined following the method described in Section 4.2.5.

(1) Reaction temperature: The reaction was carried out with 5 mmol/L hydroquinone and 20 mmol/L sucrose as substrates (prepared in phosphate buffer, pH 7.0), with 100 μL of the purified enzyme solution and phosphate buffer to make up 1 mL. The reaction was performed in the dark at 25, 30, 35, 40, 42, and 45 °C for 2 h. Other procedures followed those described in Section 4.2.8.
(2) Reaction pH: The reaction was carried out with 5 mmol/L hydroquinone and 20 mmol/L sucrose as substrates (prepared in phosphate buffer at pH 5.0, 6.0, 7.0, 8.0, 9.0, or 10.0), with 100 μL of the purified enzyme solution and phosphate buffer to make up 1 mL. The reaction was performed in the dark at 25 °C for 2 h. Other procedures followed those described in Section 4.2.8.
(3) Final hydroquinone concentration: The reaction was carried out with final hydroquinone concentrations of 2, 4, 5, 6, 8, or 10 mmol/L and 20 mmol/L sucrose as the substrate (prepared in phosphate buffer, pH 5.0), with 100 μL of the purified enzyme solution and phosphate buffer to make up 1 mL. The reaction was performed in the dark at 25 °C for 2 h. Other procedures followed those described in Section 4.2.8. Since the hydroquinone concentration was the variable in this optimization, α-arbutin yield was used as the comparison metric instead of the molar yield or hydroquinone conversion rate.
(4) Final sucrose concentration: The reaction was carried out with 6 mmol/L hydroquinone and sucrose at final concentrations of 6, 18, 30, 42, or 54 mmol/L (prepared in phosphate buffer, pH 5.0), with sucrose-to-hydroquinone ratios of 1:1, 3:1, 4:1, 5:1, 7:1, or 9:1. A total of 100 μL purified enzyme solution and phosphate buffer were added to make up 1 mL. The reaction was performed in the dark at 25 °C for 2 h. Other procedures followed those described in Section 4.2.8.
(5) Reaction time: The reaction was carried out with 6 mmol/L hydroquinone and 42 mmol/L sucrose as substrates (prepared in phosphate buffer, pH 5.0), with 100 μL of the purified enzyme solution and phosphate buffer to make up 1 mL. The reaction was performed in the dark at 25 °C for 2, 4, 6, 8, 10, and 12 h. Other procedures followed those described in Section 4.2.8.
(6) Enzyme dose: The reaction was carried out with 6 mmol/L hydroquinone and 42 mmol/L sucrose as substrates (prepared in phosphate buffer, pH 5.0), with the enzyme solution added at a volume of 50, 100, 150, or 200 μL. Phosphate buffer was added to make up 1 mL, and the reaction was performed in the dark at 25 °C for 6 h. Other procedures followed those described in Section 4.2.8.
(7) Addition of Vc: The reaction was carried out with 6 mmol/L hydroquinone and 42 mmol/L sucrose as substrates (prepared in phosphate buffer, pH 5.0), with 200 μL of the purified enzyme solution and phosphate buffer to make up 1 mL. Ascorbic acid (Vc) was added at a final concentration of 0, 0.1, 0.2, 0.3, 0.4, or 0.5 mmol/L. The reaction was performed in the dark at 25 °C for 6 h. The rest of the procedure followed that described in Section 4.2.8.

## 5. Conclusions

In this study, an ASase (CtAs) from *Calidithermus terrae* was mined and selected. The optimal conditions for its soluble expression were determined to be the addition of IPTG at a final concentration of 0.5 mmol/L to the LB medium when the OD_600_ reached 1.0, followed by incubation at 20 °C and 200 r/min for 18 h. The optimal temperature for CtAs was 42 °C, and the optimal pH was 9.5. Na^+^, K^+^, Mg^2+^, EDTA, and methanol were found to activate CtAs at both low and high concentrations. Using sucrose as the sole substrate under optimal conditions, it was observed that low sucrose concentrations favored isomerization reactions, high sucrose concentrations favored hydrolysis reactions, and polymerization reactions were largely unaffected. The optimal conditions for the enzymatic synthesis of α-arbutin were found to be 25 °C and pH 5.0, with final concentrations of 42 mmol/L sucrose and 6 mmol/L HQ as substrates (a donor-to-acceptor ratio of 7:1), 200 μL (0.2 mg/mL) of the purified enzyme, and 0.10 mmol/L Vc. The reaction was carried out in the dark for 6 h, resulting in a molar yield of α-arbutin of 62.78% and a molar conversion rate of hydroquinone of 74.60%, nearly doubling compared to the pre-optimization results.

In subsequent research, further exploration of the interactions between the active sites within its structure, nearby amino acid residues, and substrates can be conducted through methods such as homology modeling and molecular docking. Saturation mutagenesis and combinatorial mutation strategies can be employed to mutate appropriate sites, thereby enhancing its glycosyltransferase activity to better catalyze the synthesis of α-arbutin. Additionally, an exploration into improving its other enzymatic activities can be undertaken to better adapt it to industrial applications.

## Figures and Tables

**Table 1:** Screening of ASases for the enzymatic synthesis α-arbutin.

ASase	GenBank	Source Strain	Homology of Amino Acid Sequences (%)	Protein Concentration (mg/mL)	Enzyme Activity (U/mL)	Specific Enzyme Activity (U/mL)	Molar Yield of α-Arbutin (%)	Molar Conversion Rate of Hydroquinone (%)
XoAs	QBG87180	*Xanthomonas oryzae*	78.74	7.75	21.20	2.73	11.12	51.26
SaAs	PZQ29656	*Stenotrophomonas acidaminiphila*	65.61	1.07	4.78	4.47	6.01	50.04
DrAs	SEJ28255	*Deinococcus reticulitermitis*	44.59	2.56	2.57	1.00	-	-
CtAs	RIH84646	*Calidithermus terrae*	42.22	19.22	18.29	0.95	33.79	88.12
CaAs	GEO32564	*Cellulomonas aerilata*	41.80	1.04	1.32	1.27	-	-
OaAs	KPL73089	*Ornatilinea apprima*	36.39	1.80	1.32	0.73	-	-

**Table 2:** Effects of different metal ions and reagents on CtAs.

Metal Ions and Reagents	Relative Enzyme Activity (%)			
	0.2 mmol/L	1 mmol/L	5 mmol/L	10 mmol/L
Control	100.00	100.00	100.00	100.00
Na^+^	143.66 ± 2.55	130.99 ± 2.30	132.70 ± 2.56	128.81 ± 2.69
K^+^	132.36 ± 3.12	145.64 ± 1.87	148.98 ± 0.26	142.44 ± 2.64
Mg^2+^	138.49 ± 1.44	138.69 ± 1.78	123.37 ± 3.21	118.26 ± 1.32
Ca^2+^	95.57 ± 1.36	66.01 ± 1.12	59.54 ± 0.54	49.32 ± 1.13
Zn^2+^	62.74 ± 0.82	58.92 ± 1.49	47.96 ± 0.78	46.66 ± 0.56
Ba^2+^	78.75 ± 2.61	65.80 ± 1.19	55.45 ± 1.01	60.35 ± 1.78
Mn^2+^	122.28 ± 2.48	131.34 ± 4.10	57.29 ± 1.60	65.46 ± 1.60
Ni^2+^	64.44 ± 1.28	56.61 ± 1.13	54.70 ± 0.51	56.47 ± 1.46
Cu^2+^	130.99 ± 0.31	159.60 ± 4.86	56.06 ± 1.79	69.89 ± 2.10
Fe^2+^	147.28 ± 1.91	147.48 ± 2.97	104.36 ± 1.28	35.76 ± 1.30
EDTA	150.26 ± 0.34	133.88 ± 0.76	119.86 ± 0.22	107.01 ± 0.62
SDS	135.52 ± 1.01	154.65 ± 0.56	48.62 ± 0.95	98.36 ± 4.94

Values are the means of three replicates ± standard deviations.

**Table 3:** Effects of different organic solvents on CtAs.

Organic Solvents	Relative Enzyme Activity (%)			
	1%	5%	10%	20%
Control	100.00	100.00	100.00	100.00
Methanol	129.42 ± 0.98	108.98 ± 1.33	119.79 ± 0.84	107.34 ± 0.41
Ethanol	121.30 ± 0.75	114.42 ± 0.93	113.50 ± 0.67	90.50 ± 1.06
n-Butanol	129.69 ± 0.43	111.01 ± 1.11	63.43 ± 1.27	66.06 ± 0.43
Acetonitrile	111.07 ± 1.75	97.05 ± 1.09	90.43 ± 0.28	52.88 ± 1.02
Dimethyl sulfoxide	101.44 ± 0.41	103.54 ± 0.60	101.18 ± 0.34	91.35 ± 0.88

Values are the means of three replicates ± standard deviations.

**Table 4:** Enzymatic properties of recombinant ASases from different bacterial sources.

Source Strain	Optimal Temperature (°C)	Optimal pH	Percentage (%)			References
			Polymerization Reaction	Isomerization Reaction	Hydrolysis Reaction	
*Calidithermus terrae*	42	9.5	32.3	44.9	22.8	This study
*Neisseria polysaccharea*	35	7.0	NR	NR	NR	[29]
*Arthrobacter chlorophenolicus*	NR	8.0	78.4	19.7	1.9	[3]
*Alteromonas macleodii*	45.0	8.0	NR	NR	NR	[3]
*Cellulomonas carboniz T26*	40.0	7.0	84.0	10.2	5.8	[20]
*Deinococcus geothermalis*	45.0	8.0	71.0	22.0	7.0	[30,31]
*Deinococcus radiodurans*	50.0	8.0	56.9	33.5	9.6	[32,33]
*Deinococcus radiopugnans*	40–45	8.0	82.7	11.5	5.8	[34]
*Methylotuvimicrobium alcaliphilum*	30.0	8.0	NR	NR	NR	[35]
*Methylobacillus flagellatus*	45.0	8.5	75.5	15.0	9.5	[36]
*Neisseria polysaccharea*	37.0	8.0	80.1	14.5	5.4	[37,38]
*Neisseria subflava*	45.0	8.0	NR	NR	NR	[39]
*Synechococcus* sp.	30.0	6.5–7.0	NR	NR	NR	[40]

NR: not reported.

**Table 5:** Oligonucleotides used for PCR.

Primer	Sequence
XoAS-F	5′ CGCCA TATGAGCACCTCCCCGATCGAT 3′
XoAS-R	5′ CCGC TCGAGGGCACCGCGCTGCAACCA 3′
SaAS-F	5′ CGCCA TATGAATCCTGCGATCGAGACG 3′
SaAS-R	5′ CCGC TCGAGCTGAGCGGACTTGCCCCG 3′
DrAS-F	5′ CGCCA TATGCTGTGGCCTGTGTTG 3′
DrAS-R	5′ CCGG AATTCTTAATGGTGATGGTGATGATG GTCATCCCGGCTCACCAGCC 3′
CtAS-F	5′ CGCCA TATGTTCTCCACCCCGCTC 3′
CtAS-R	5′ CCGG AATTCTTAATGGTGATGGTGATGATG CACCTCATCCGTGATCCACA 3′
CaAS-F	5′ CGCCA TATGAGCGGCGCCTTCCCC 3′
CaAS-R	5′ CCGG AATTCTTAATGGTGATGGTGATGATG TCCGGGCTCCGGCGTGACCC 3′
OaAS-F	5′ CGCCA TATGGAAAAAGCCATCCATTAC 3′
OaAS-R	5′ CCGC TCGAGGGCGCGAAGAATCAGGAA 3′

Note: Underlined sequences are the restriction sites (upstream primer: *Nde* I; downstream primer: *Xho* I/*Eco*R I).

## Data Availability

All relevant data are included within the article.
